# Transplanted astrocytes derived from BMP- or CNTF-treated glial-restricted precursors have opposite effects on recovery and allodynia after spinal cord injury

**DOI:** 10.1186/jbiol85

**Published:** 2008-09-19

**Authors:** Jeannette E Davies, Christoph Pröschel, Ningzhe Zhang, Mark Noble, Margot Mayer-Pröschel, Stephen JA Davies

**Affiliations:** 1 Department of Neurosurgery, Anschutz Medical Campus, University of Colorado Denver, 12800 East 19th Ave, Aurora, CO 80045, USA; 2 Department of Biomedical Genetics, University of Rochester Medical Center, 601 Elmwood Avenue, Rochester, NY 14642, USA

## Abstract

**Background:**

Two critical challenges in developing cell-transplantation therapies for injured or diseased tissues are to identify optimal cells and harmful side effects. This is of particular concern in the case of spinal cord injury, where recent studies have shown that transplanted neuroepithelial stem cells can generate pain syndromes.

**Results:**

We have previously shown that astrocytes derived from glial-restricted precursor cells (GRPs) treated with bone morphogenetic protein-4 (BMP-4) can promote robust axon regeneration and functional recovery when transplanted into rat spinal cord injuries. In contrast, we now show that transplantation of GRP-derived astrocytes (GDAs) generated by exposure to the gp130 agonist ciliary neurotrophic factor (GDAs^CNTF^), the other major signaling pathway involved in astrogenesis, results in failure of axon regeneration and functional recovery. Moreover, transplantation of GDA^CNTF ^cells promoted the onset of mechanical allodynia and thermal hyperalgesia at 2 weeks after injury, an effect that persisted through 5 weeks post-injury. Delayed onset of similar neuropathic pain was also caused by transplantation of undifferentiated GRPs. In contrast, rats transplanted with GDAs^BMP^ did not exhibit pain syndromes.

**Conclusion:**

Our results show that not all astrocytes derived from embryonic precursors are equally beneficial for spinal cord repair and they provide the first identification of a differentiated neural cell type that can cause pain syndromes on transplantation into the damaged spinal cord, emphasizing the importance of evaluating the capacity of candidate cells to cause allodynia before initiating clinical trials. They also confirm the particular promise of GDAs treated with bone morphogenetic protein for spinal cord injury repair.

## Background

Two critical challenges that must be addressed in the development of cell-based tissue repair strategies are the identification of optimal cell types and the identification of instances in which cell transplantation may create severe adverse side effects. The first problem is important because of the considerable resources that will be required to establish clinical efficacy of putative treatments. The second problem is perhaps of even greater importance, because adverse outcomes in clinical trials could seriously hinder the development of stem cell technology for tissue repair.

Diseases of the central nervous system (CNS) are of particular interest as candidates for clinical evaluation of cell transplantation therapies, with the treatment of spinal cord injury being one of the primary targets for early translation of laboratory efforts to clinical trials. A variety of cell types of both non-CNS and CNS origin, such as Schwann cells [1], olfactory ensheathing glia [2], marrow stromal cells [3,4] and oligodendrocyte progenitor cells [5], are being considered for clinical trial to treat spinal cord injuries. One of the most attractive reasons for considering the use of non-CNS cells such as Schwann cells, olfactory ensheathing cells and marrow stromal cells for CNS repair has been their relative ease of isolation compared to cells of CNS origin. However, continuing advances in stem cell technology are making the goal of utilizing CNS cell types to repair the injured CNS more readily attainable.

One new potential candidate for use in CNS repair is a population of astrocytes that is derived by treatment of glial progenitor cells (GRPs) of the embryonic spinal cord with bone morphogenetic protein (BMP) before transplantation. We call this astrocyte population GDAs^BMP^. The replacement of damaged neurons and oligodendrocytes in the injured or diseased spinal cord has been pursued by a number of laboratories (reviewed in [6]), but less attention has been given to the development of astrocyte replacement therapies, despite the fact that astrocytes account for the majority of cells in the adult CNS [7] and are critical to normal CNS function [8]. This relative lack of attention is probably due to the modest levels of axon regeneration and lack of functional recovery seen after transplantation into the injured CNS of astrocytes isolated from the immature cortex [9-12]. Factors such as contamination with microglia and undifferentiated progenitors, isolation from cortex rather than spinal cord, and a phenotype that is less supportive of axon growth (resulting from the prolonged *in vitro *growth required to generate postnatal astrocyte cultures) [13], may have rendered these glial cultures suboptimal for repairing the injured adult spinal cord.

In contrast to the lack of effect of astrocyte transplantation in previous studies, GDAs^BMP ^promote robust axon regeneration, neuroprotection and functional recovery after acute spinal cord injury [14]. The ability to generate specific subtypes of astrocytes from defined glial precursors provides a new platform for the development of astrocyte-based transplantation therapies for the injured adult CNS. Transplantation of GDAs^BMP ^to acute transection injuries of adult rat spinal cord promoted first, a 39% efficiency of endogenous ascending dorsal column axon regeneration across sites of injury; second, protection of axotomized red nucleus neurons; third, a significant reduction of inhibitory scar formation; and fourth, a degree of behavioral recovery from dorsolateral funiculus injuries that enabled rats to generate an average score by 4 weeks after transplantation that was statistically indistinguishable from that obtained for uninjured animals on a stringent test of volitional foot placement [14]. Moreover, this strategy allows the rapid generation of astrocytes directly from embryonic precursor cells, thus eliminating the use of the prolonged *in vitro *purification procedures that result in a phenotype that is less supportive of axon growth [13].

Recent studies demonstrating the ability of transplanted neuroepithelial stem cells (NSCs) to cause pain syndromes in animals with spinal cord injury have, however, raised concerns that the astrocytes generated by transplanted stem or progenitor cells might cause adverse effects that outweigh any benefits. Two recent studies have shown that transplantation of NSCs into acute spinal cord injuries in rats promotes the onset of both mechanical allodynia (a painful response to normally non-painful touch stimuli) and thermal hyperalgesia (abnormal sensitivity to heat) [15,16]. These adverse side effects correlated with the differentiation of the transplanted NSCs into astrocytes, and were prevented by the suppression of astrocyte generation by overexpression of the transcription factor neurogenin-2 in the transplanted NSCs [15]. It was therefore very important to determine whether transplantation of astrocytes, or of precursor cells capable of generating astrocytes, would promote the onset of allodynia, or whether this is a problem unique to the transplantation of NSCs.

**Figure 1:**
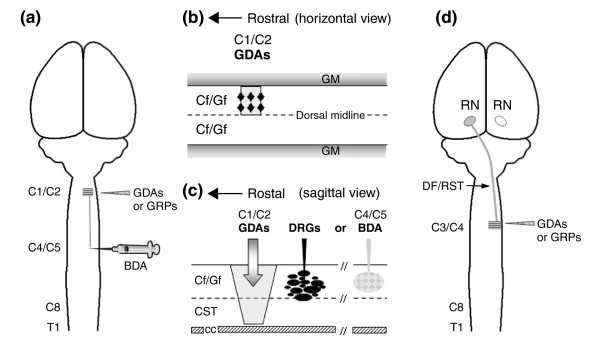
Schematic illustration of the adult rat models of spinal cord injury used in this study. **(a) **Dorsal view of rat brain and spinal cord. Dorsal column white matter on the right side was transected (shaded area) at the C1/C2 spinal level, and the ability of either BDA-labeled endogenous axons or axons from microtransplanted GFP-expressing adult sensory neurons (DRGs) to cross injuries bridged with GDAs or GRPs was assayed. **(b) **Horizontal and **(c) **sagittal views of the dorsal column white-matter pathways at the C1/C2 cervical vertebrae of the spinal cord. (b) Injections of GDAs or GRPs (black diamonds) suspended in medium were made directly into the centers of the injury sites as well as their rostral and caudal margins in the cervical spinal cord. **(c) **A discrete population of endogenous ascending axons within the cuneate and gracile white-matter pathways of dorsal columns was labeled by BDA injection at the C4/C5 spinal level (5 mm caudal to the injury site, shaded). Alternatively, microtransplants of GFP^+ ^DRGs were injected 500 μm caudal to the injury site. **(d) **The right-side dorsolateral funiculus white matter containing descending axons of the rubrospinal tract was transected at the C3/C4 spinal level and GDAs or GRPs were transplanted as described for dorsal column injuries. CC, central canal; Cf, cuneate fasciculus; CST, corticospinal tract; DF, dorsolateral funiculus; Gf, gracile fasciculus; GM, gray matter; RN, red nucleus; RST, rubrospinal tract; T1, level of the first thoracic vertebra.

The study reported here was carried out to determine whether all astrocytes generated from GRPs [17] were equally able to promote repair of adult injured spinal cord. Two types of astrocytes can be generated from embryonic spinal GRPs – GDAs^BMP ^and GDAs^CNTF ^(astrocytes derived from the gp130 receptor agonist ciliary neurotrophic factor (CNTF)). We found that transplantation of these two types of astrocytes into acute spinal cord injuries (Figure 1) yielded significantly different outcomes. In contrast to GDAs^BMP^, we found that GDAs^CNTF ^provided no benefit and, more importantly, transplantation of either GDAs^CNTF ^or undifferentiated GRPs caused neuropathic pain. Our results also confirm earlier work [14] showing that transplantation of GDAs^BMP ^generated by controlled pre-differentiation of GRPs can provide substantial benefits after spinal cord injury and that this pre-differentiation can avoid the problem of transplanted glial precursors themselves causing pain syndromes.

## Results

### Characterization of GDAs *in vitro*

GRPs exposed to BMP-4 generate astrocytes (GDAs^BMP^) with a flat, type-1 antigenic phenotype that express glial fibrillary acidic protein (GFAP) and do not label with the A2B5 antibody [14]. In contrast, GRPs grown in the presence of the gp130 receptor agonist CNTF generate GFAP^+ ^astrocytes (GDAs^CNTF^) with processes that are labeled by A2B5 [17]. In seeking to use GDAs for repairing the injured spinal cord, it is critical to know whether the favorable properties of GDAs^BMP ^are solely a reflection of the embryonic age and/or identity of the glial precursor cell from which they are derived, or whether it is necessary to generate a very specific population of astrocytes from these precursor cells to promote repair.

**Figure 2:**
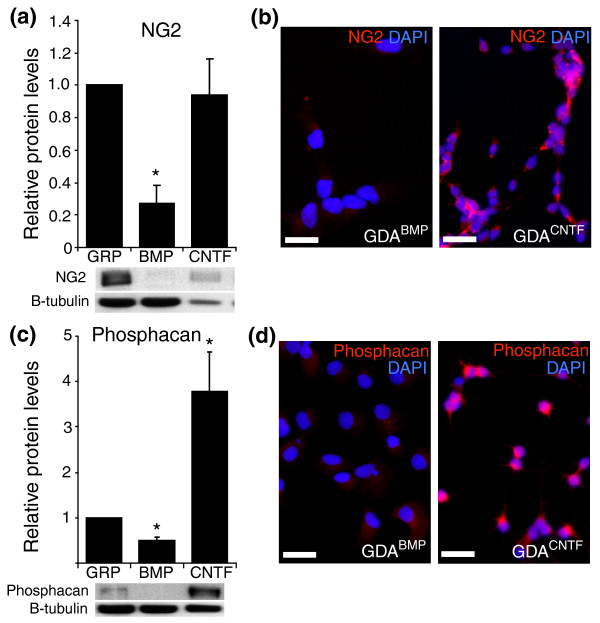
GDAs^BMP^, GDAs^CNTF ^and GRPs express different levels of NG2 and phosphacan *in vitro*. GRPs were induced to differentiate into astrocytes by exposure to BMP or CNTF. Relative levels of expression of NG2 and phosphacan proteins were determined by quantitative Western blot and immunocytochemical analysis. **(a,c) **Western blot analysis of whole-cell lysates demonstrates that GDAs^CNTF ^express higher levels of (a) NG2 and (c) phosphacan. The graph shows fold change in protein levels for GDAs compared to GRPs. Error bars represent 1 standard deviation (SD). *p < 0.05. **(b,d) **Immunofluorescent labeling of cells using (b) anti-NG2 antibodies and (d) anti-phosphacan. Scale bars 50 μm.

**Figure 3:**
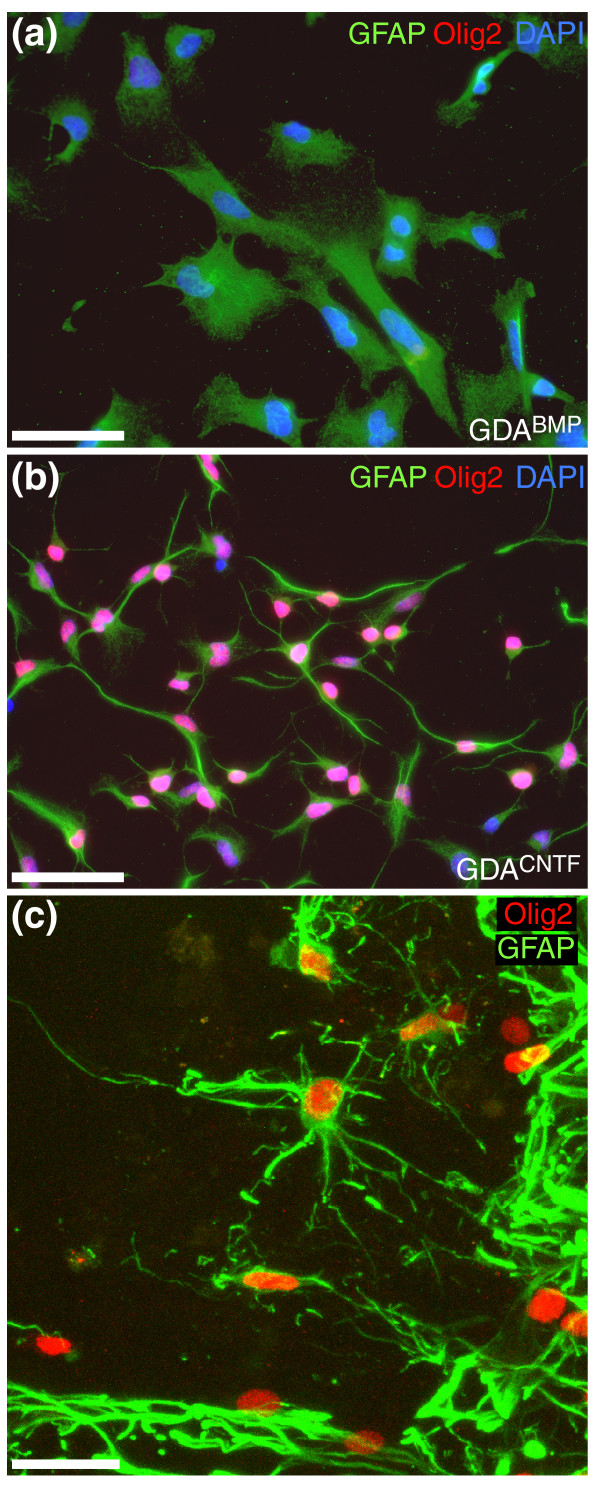
Differential expression of Olig2 protein by different astrocyte populations. **(a) **GDAs^BMP ^do not express Olig2. **(b) **In sharp contrast, GDAs^CNTF ^are uniformly immunopositive for Olig2 *in vitro*. **(c) **A subset of endogenous GFAP^+ ^astrocytes in the margins of untreated dorsal column spinal cord injuries is also Olig2-immunoreactive. Survival, 8 days post-injury. Note the nuclear localization of Olig2 in GDAs^CNTF ^*in vitro *and in reactive, endogenous GFAP^+ ^astrocytes *in vivo*. Scale bars: (a,b) 50 μm; (c) 25 μm.

To answer this question, we first characterized GDAs^BMP ^and GDAs^CNTF ^*in vitro *and found that GDAs^CNTF ^had properties suggesting they would be less suitable than GDAs^BMP ^for repairing the injured adult CNS. Compared with GDAs^BMP^, GDAs^CNTF ^express elevated levels of the axon-growth-inhibitory chondroitin sulfate proteoglycans (CSPGs) NG2 (Figure 2a,b) and phosphacan (Figure 2c,d), both of which are also expressed at high levels in glial scar tissue [18]. We also found that GDAs^BMP ^and GDAs^CNTF ^cells differed in their regulation of the transcriptional regulator Olig2 *in vitro *(Figure 3a,b). In agreement with previous observations of the effects of BMP on Olig2 expression in cortical neural progenitors [19], GRPs exposed to BMP-4 downregulated Olig2 expression (Figure 3a). In contrast, GDAs^CNTF ^had high levels of Olig2 in their nuclei (Figure 3b). Several recent studies have reported the natural generation of cells that coexpress Olig2 and GFAP *in vivo *after injury to the brain [20,21]. Although those studies described cytoplasmic rather than nuclear localization of Olig2, our examination of control injured spinal cords at 8 days revealed the presence of endogenous GFAP^+ ^cells with nuclear localization of Olig2 (Figure 3c).

### Characterization of transplanted GDAs^CNTF ^*in vivo*

**Figure 4:**
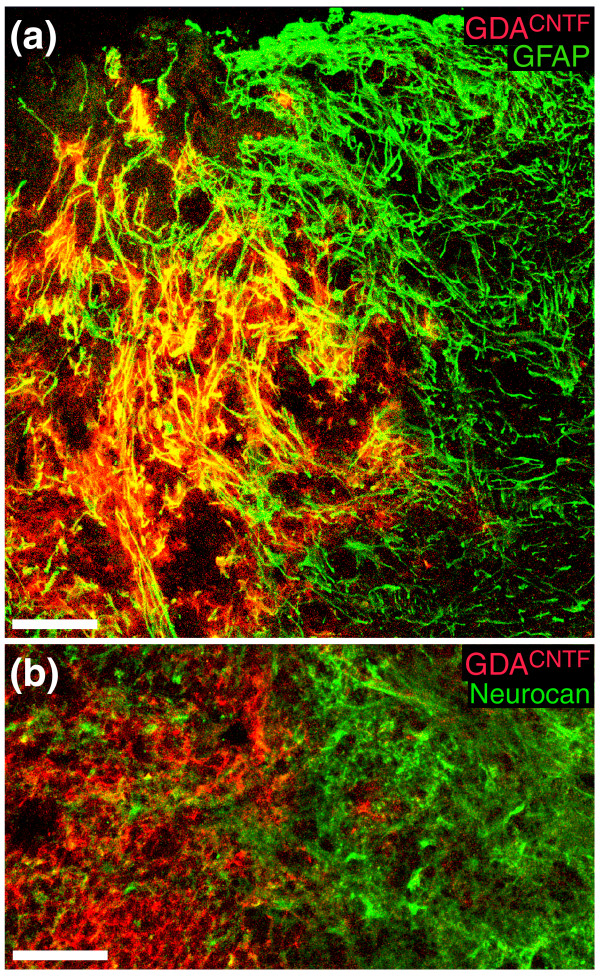
GDAs^CNTF ^express GFAP and neurocan after transplantation into spinal cord injuries. **(a) **Intra-injury GDAs^CNTF ^are uniformly GFAP^+ ^within acute dorsal column injuries. Note the co-localization (yellow) of human placental alkaline phosphatase (hPAP, red) with GFAP (green). GDAs^CNTF ^have also failed to align host astrocytic processes within injury margins. Survival, 8 days post-injury/transplantation. **(b) **High-magnification confocal image of neurocan immunoreactivity at the injury margin and within a GDA^CNTF^-transplanted injury site at 8 days after injury/transplantation. Note that some GDAs^CNTF ^are immunoreactive for neurocan (green). In contrast, intra-injury transplanted GDAs^BMP ^(not shown) do not express GFAP or neurocan, and can align host astrocytic processes within injury margins [14]. Scale bars 100 μm.

**Figure 5:**
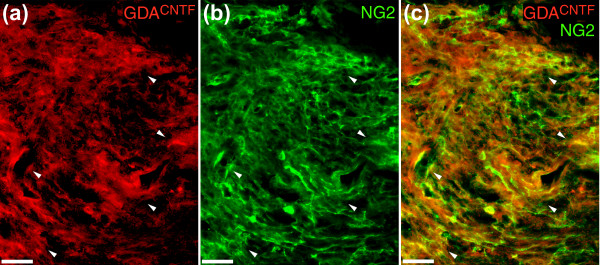
NG2 immunoreactivity in GDA^CNTF^-transplanted dorsal column injuries. **(a) **Transplanted hPAP^+ ^GDAs^CNTF ^(arrowheads) at 8 days post injury/transplantation. **(b) **The same slide stained for NG2 (green) showing that the transplanted cells (arrowheads) show immunoreactivity for NG2. **(c) **Co-localization (yellow) of NG2 and hPAP immunoreactivity in regions containing higher densities of GDAs^CNTF ^(arrowheads). In general, regions of the injury site that contained higher densities of hPAP^+ ^GDAs^CNTF ^had a higher density of NG2 immunoreactivity. Scale bars 50 μm.

**Figure 6:**
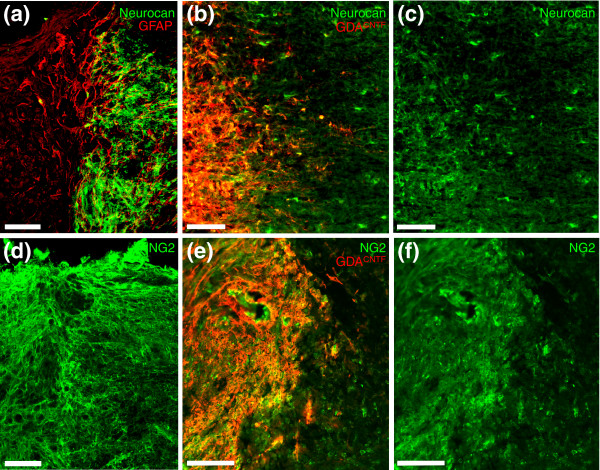
Transplanted GDAs^CNTF ^express neurocan and NG2, but suppress host expression of these two CSPGs at 4 days post-injury/transplantation. **(a) **At 4 days after injury, control dorsal column injury margins express dense neurocan immunoreactivity (green) mainly associated with GFAP^- ^processes. Note the absence of neurocan immunoreactivity in the injury center (to the left). **(b,c) **While neurocan immunoreactivity in host white matter was markedly lower and mainly associated with astrocyte cell bodies, many intra-injury GDAs^CNTF ^within injury centers displayed neurocan immunoreativity. **(d) **NG2 immunoreactivity in control injuries is high in both injury centers and margins. **(e,f) **Although overall levels of NG2 immunoreactivity were reduced within injury centers and margins of GDA^CNTF^-transplanted injury sites compared to untreated control injuries (compare (d) and (f)), levels of NG2 immunoreactivity were still higher than that previously observed for identical dorsal column injuries transplanted with GDAs^BMP ^[14]. Scale bars 200 μm.

**Figure 7:**
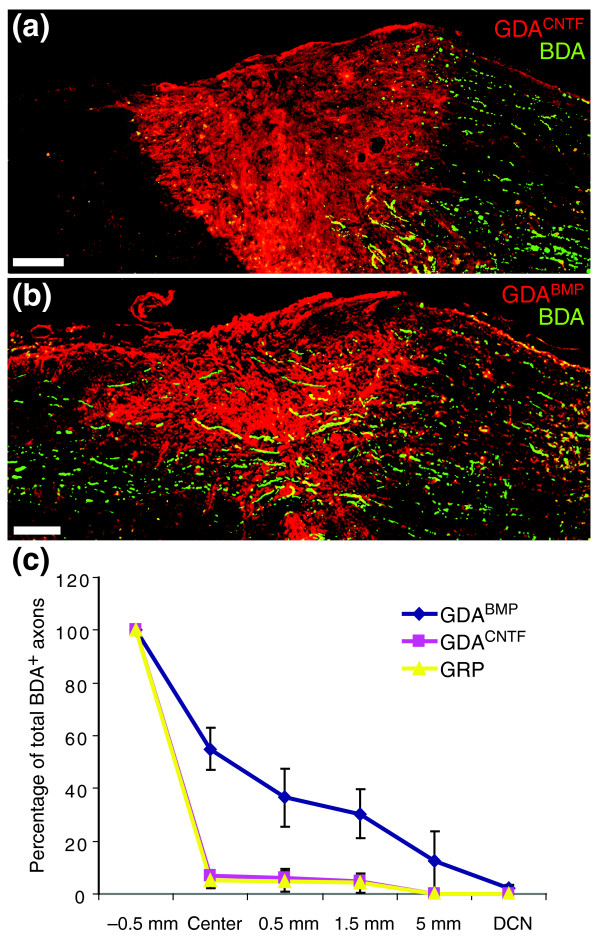
Failure of axons to regenerate across GDA^CNTF ^or GRP transplanted dorsal column injuries. **(a) **Biotinylated dextran amine (BDA)-labeled endogenous, ascending dorsal column axons (green) fail to cross GDA^CNTF^-transplanted injury sites and instead form dystrophic endings within caudal injury margins. While a few axons sprout towards the injury center, BDA^+ ^axons are rarely detected beyond the injury/transplantation site at 8 days post-injury/transplantation. Scale bar 200 μm. **(b) **In contrast, transplanted GDAs^BMP ^support extensive axon growth across dorsal column injuries at 8 days after injury/transplantation. Scale bar 200 μm. **(c) **Quantification of numbers of regenerating BDA^+ ^axons in GDA- or GRP-transplanted dorsal column white matter at 8 days after injury and transplantation. BDA-labeled axons were counted in every third sagittally oriented section within the injury center and at points 0.5 mm, 1.5 mm and 5 mm rostral to the injury site and within the dorsal column nuclei (DCN). Note that 55% of BDA^+ ^axons reached the centers of GDA^BMP^-transplanted injuries, and 36% to 0.5 mm beyond the injury site. After GDA^CNTF ^or GRP transplantation, however, only 7% and 5.3% of BDA^+ ^axons, respectively, were observed within injury centers, with only 4.6% and 4.2% of the axons observed at 0.5 mm beyond the injury site. No BDA^+ ^axons were detected beyond 1.5 mm rostral to the injury site in GDA^CNTF^- or GRP-transplanted spinal cords. Error bars represent 1 SD.

Transplanted GDAs^CNTF ^exhibited good survival and were able to completely span sites of injury (Figures 4, 5, 6, 7). We found that transplanted GDAs^CNTF ^displayed phenotypes markedly different from those previously observed for transplanted GDAs^BMP^. The majority of GDAs^CNTF^ retained their GFAP immunoreactivity after transplantation to acute spinal cord injury, particularly for those cells adjacent to injury margins (Figure 4a). Subsets of intra-injury GDAs^CNTF ^also displayed immunoreactivity for the axon-growth-inhibitory proteoglycan neurocan at 4 and 8 days post-injury (Figure 4b) and the majority of GDAs^CNTF ^had retained their *in vitro *immunoreactivity for NG2 (Figure 5). In contrast, our previous studies showed that GDAs^BMP ^did not retain GFAP immunoreactivity after transplantation to identical acute spinal cord injuries [14]. More importantly, transplanted GDAs^BMP ^within the center of the injured site remained negative for neurocan and NG2 immunoreactivity at 8 days after transplantation [14].

### Effects of GDAs^CNTF ^and GRPs on scar formation

Transplanted GDAs^CNTF ^and GDAs^BMP ^also had substantially different effects on the reactivity of host astrocytes at sites of injury. We previously showed that transplantation of GDAs^BMP ^suppressed the gliotic response of host astrocytes within injury margins and promoted a remarkable linearization of their processes [14]. Transplantation of GDAs^CNTF^, in contrast, did not suppress astrogliosis, nor did these cells align host astrocytes in injury margins. Instead, the margins of GDA^CNTF^-transplanted injury sites contain a meshwork of misaligned, hypertrophic GFAP^+ ^astrocytic processes (Figure 4a), similar to that observed in both control untreated injuries and the margins of GRP-transplanted injuries [14]. GDA^CNTF ^and GRP transplantation did, however, result in a suppression of neurocan and NG2 expression by host tissue at sites of injury at 4 days post-injury, an effect we previously observed following transplantation of GDAs^BMP ^[14]. At 4 days after injury, the margins of control, untreated injuries displayed a high density of neurocan immunoreactivity (Figure 6a) associated with numerous fine GFAP^- ^processes that we previously showed to be associated with NG2^+^ glia [18]. In contrast, at 4 days after injury and transplantation of GDAs^CNTF ^(Figure 6b,c) or GRPs (Additional data file 1), neurocan immunoreactivity within injury margins was mainly associated with the cell bodies of GFAP^+ ^host white-matter astrocytes, a pattern of expression similar to that observed for neurocan at 2 days after injury in untreated control animals [18]. However, by 8 days after injury and GDA^CNTF ^transplantation, neurocan and NG2 immunoreactivity at sites of injury was similar in intensity and distribution to that seen in untreated control injuries (Figures 4b and 5b). Thus, like GDAs^BMP ^transplanted to acute spinal cord injuries, GRPs and GDAs^CNTF ^had promoted transient suppression of axon-growth-inhibitory CSPGs by host tissues; but unlike GDAs^BMP^, neither GDAs^CNTF ^nor GRPs [14] suppressed astrogliosis or aligned host astrocytes within injury margins.

### GDAs^CNTF ^do not support axon regeneration *in vivo*

We next examined the ability of GDAs^CNTF ^to promote axon regeneration *in vivo*, both of endogenous ascending dorsal column axons and of axons emanating from transplanted adult dorsal root ganglion (DRG) neurons. For analysis of endogenous axon regeneration, a discrete population of ascending axons aligned with the injury site was traced with a single injection of biotinylated dextran amine (BDA) at a distance 6 mm caudal to GDA^CNTF^-, GDA^BMP^-, or GRP-transplanted or control transection injuries of the right-hand dorsal column cuneate and gracile white-matter pathways. This minimized the labeling of spared axons. Previous studies have shown that around 30–40% of ascending dorsal column axons projecting to the dorsal column nuclei arise from postsynaptic dorsal column neurons in spinal lamina IV and that 25% of ascending dorsal column axons are also propriospinal in origin [22,23]. It has been shown that only 15% of primary afferents of DRG neurons entering the spinal cord at lumbar levels reach the cervical spinal cord and that most leave dorsal column white matter within two to three segments of entering [24]. Therefore, our *en passage *labeling of dorsal column axons at the cervical level would have included significant proportions of axons from both CNS spinal neurons and DRG neurons. To further test the ability of transplanted GDAs^CNTF ^to support axon growth across an acute spinal cord injury in a model that eliminates the possibility of axon sparing, we examined their ability to support the growth of adult sensory axons across identical stab injuries in an adult DRG neuron/GDA transplant spinal cord injury model [14]. In these experiments, a separate series of animals received microtransplants of adult mouse sensory neurons labeled with green fluorescent protein (GFP) acutely into dorsal column white matter at a distance of 400–500 μm caudal to GDA^CNTF^-transplanted injuries (Figure 1c).

Transplantation of either GRPs or GDAs^CNTF ^to acute dorsal column transection injuries failed to improve the regeneration of endogenous ascending dorsal column axons above that observed in untreated injuries (Figure 7). There was also a complete failure of axons grown from adjacent microtransplanted adult mouse DRG neurons expressing enhanced green fluorescent protein (EGFP) to cross GDA^CNTF^-transplanted injuries (Additional data file 2). In both experimental models, the majority of axons instead formed dystrophic endings within the caudal injury margins of GDA^CNTF^-transplanted injuries, (Figure 7a and Additional data file 2a), an axon morphology well known as the hallmark of failure of axon regeneration in CNS injury [25,26]. Quantitative analysis of the efficiency of ascending dorsal column axon regeneration in GDA^CNTF^- and GRP-transplanted rats at 8 days after transplantation/injury showed that only 7% (SD ± 2.0) and 5.3% (SD ± 3.0), respectively, of BDA-labeled axons within white matter 0.5 mm caudal to injury sites had reached injury centers; 6.2% (SD ± 3.5) and 4.7% (SD ± 3.9) of axons had extended 0.5 mm beyond injury sites into distal white matter, with 4.6% (SD ± 2.3) and 4.2% (SD ± 3.6) reaching 1.5 mm beyond injury sites (Figure 7c). No BDA-labeled axons were detected beyond 1.5 mm in distal white matter or within the dorsal column nuclei of both GDA^CNTF^- and GRP-transplanted rats (Figure 7c). All the percentages of BDA-labeled axons within injury sites and at all points beyond were not statistically different from those quantified for BDA-labeled endogenous ascending dorsal column axons in identical control, untransplanted injuries [14] (ANOVA, *p *> 0.05).

The failure of both GDAs^CNTF ^and GRPs (see also [14]) to support axon regeneration is in stark contrast to the ability of transplanted GDAs^BMP^ to promote regeneration of endogenous dorsal column axons across spinal cord injuries. In GDA^BMP^-transplanted animals, 55% (SD ± 8.0) of labeled axons extended to the injury center, 36.5% (SD ± 11.0) extended to 0.5 mm beyond the injury site, and 30.4% (SD ± 9.2) had extended to 1.5 mm beyond the injury site (Figure 7c). Furthermore, 12.6% (SD ± 9.0) of labeled axons were detected within white matter at 5 mm beyond the injury site, and 2.1% (SD ± 1.4) were observed within the dorsal column nuclei (Figure 7c). This is consistent with our previous finding that intra-injury transplants of GDA^BMP ^cells promote regeneration of 60% (SD ± 11.0) of labeled endogenous ascending dorsal column axons into the center of injury sites, and more than two-thirds of these axons were within white matter beyond the injury site by 8 days after transplantation/injury [14].

### Failure of GDAs^CNTF ^to promote locomotor functional recovery after spinal cord injury

To make a direct comparison of the ability of GDAs^CNTF ^and GDAs^BMP^ to promote functional recovery following dorsolateral funiculus transection injuries to the spinal cord, an analysis of grid-walk performance for GDA^CNTF^- and GDA^BMP^-transplanted rats versus rats injected with control medium was carried out at times ranging from 3 to 28 days after injury/transplantation. Transection of the dorsolateral funiculus severs descending supraspinal axons and results in chronic deficits in both fore-and hindlimb motor function [27] that can be detected by the grid-walk behavioral test [28]. We have previously shown that transplantation of GDAs^BMP ^into acute dorsolateral funiculus injuries resulted in robust improvements in grid-walk locomotor function compared to media-injected control injured animals at all time points ranging from 3 to 28 days post-injury. In contrast, transplantation of undifferentiated GRPs failed to improve scores to greater than those observed for control injured animals [14].

**Figure 8:**
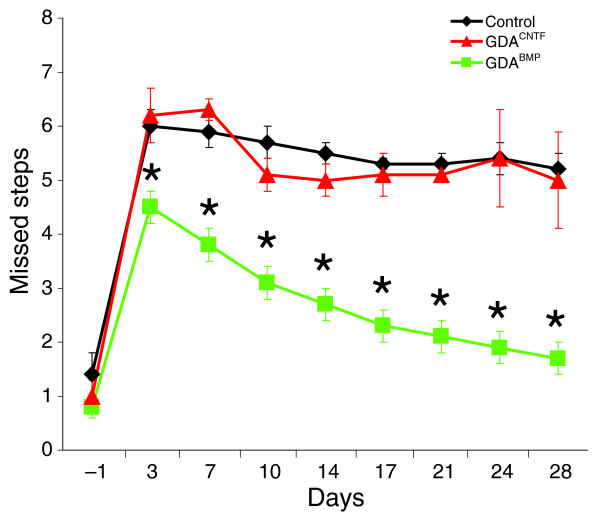
Grid-walk analysis of locomotor recovery. Graph showing the average number of missed steps per experimental group from 1 day before injury (baseline pre-injury) to 28 days after injury for all GDA-transplanted/dorsolateral funiculus injured rats versus the control-injured animals. GDA^BMP^-transplanted animals (green) performed significantly better than GDA^CNTF^-transplanted animals and injured control animals at all post-injury time points (*p *< 0.05). Note that the performance of GDA^CNTF^-transplanted animals was not different from untreated control injured rats at all time points (two-way repeated measures ANOVA, *p < 0.05). *N* = 9 rats per group.

Animals that received GDA^CNTF ^transplants or injections of medium alone (controls) made an average of 6.2 (SD ± 0.5) and 6.0 (SD ± 0.3) mistakes, respectively, at 3 days after injury/transplantation and showed no statistically significant improvement at any later time point, with an average of 5.2 (SD ± 0.3) and 5.0 (SD ± 0.9) mistakes at 28 days post-injury (Figure 8). Thus, despite receiving transplants of astrocytes derived from embryonic GRP cells, GDA^CNTF^-transplanted rats did not show any recovery of locomotor function when compared with controls. In contrast, at 3 days after injury/transplantation, animals receiving GDAs^BMP ^were already making an average of 4.5 (SD ± 0.3) mistakes; that is, significantly fewer than GDA^CNTF^-transplanted or control animals (Figure 8). Consistent with our previous report [14], animals receiving GDA^BMP ^transplants in the current study continued to improve significantly between 3 and 28 days after injury (two-way repeated measures ANOVA, *p *< 0.05). At 28 days after injury, GDA^BMP^-treated rats made an average of just 1.7 (SD ± 0.3) mistakes on the grid walk apparatus (Figure 8), a score that was statistically indistinguishable from their pre-injury baseline scores.

### Transplantation of GDAs^CNTF ^or GRPs fails to suppress atrophy of red nucleus neurons

**Figure 9:**
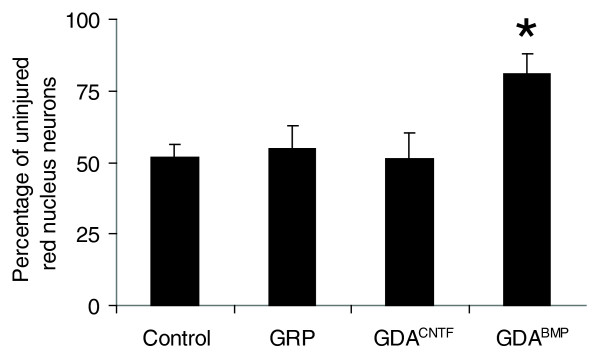
Neuroprotection of red nucleus neurons. Injured left-side red nuclei contained an average of 52% of the neurons counted in uninjured right-side red nuclei at 5 weeks after transection of the right-side rubrospinal tract. The numbers of neurons in the injured left-side red nuclei of GRP- and GDA^CNTF^-transplanted animals were no different from controls, and contained an average of 55% and 51%, respectively, of the neurons counted in the uninjured right-side nuclei. In contrast, the number of neurons in the injured left-side red nuclei of GDA^BMP^-transplanted animals was 81% of the total number of neurons in uninjured right-side nuclei. *p < 0.01. Error bars represent 1 SD.

Transection of axons of the rubrospinal tract (RST) in the dorsolateral funiculus of the spinal cord causes atrophy of significant numbers of red nucleus neurons, a process that begins approximately 1 week after spinal cord injury [29]. In the absence of intervention, the number of neurons with a cell-body diameter greater than 20 μm in the injured left-side red nucleus in control, untreated RST-injured animals fell to 52% (SD ± 4.2%) of the values in the uninjured right-side nucleus at 5 weeks after injury (Figure 9).

Consistent with our previous findings [14], animals that had received intra-spinal cord injury transplants of GDAs^BMP ^(Figure 9) once again showed a significant suppression of red nucleus neuron atrophy with 82% (SD ± 6.1) of neurons in the injured left-side red nucleus having cell body diameters greater than 20 μm when normalized to the uninjured right-side nucleus (Figure 9). In contrast, transplantation of GDAs^CNTF ^or undifferentiated GRPs into identical dorsolateral funiculus injuries completely failed to suppress neuron atrophy in the injured left-side red nucleus (Figure 9; see also Additional data file 3). Counts of neurons with a cell-body diameter greater than 20 μm in the injured left-side red nucleus in GDA^CNTF^- or GRP-treated animals were only 51% (SD ± 8.7%) and 55% (SD ± 8.0%), respectively, of the values in uninjured right-side red nucleus at 5 weeks after injury and did not differ statistically from untreated injured animals (ANOVA, *p *< 0.05). Thus, despite the fact that GDAs^BMP ^and GDAs^CNTF ^are both astrocytes derived from the same embryonic precursor cells, they do not share the same ability to rescue red-nucleus neuronal populations from atrophy.

### GDAs^CNTF ^or GRPs, but not GDAs^BMP^, induce mechanical allodynia and thermal hyperalgesia when transplanted into sites of spinal cord injury

**Figure 10:**
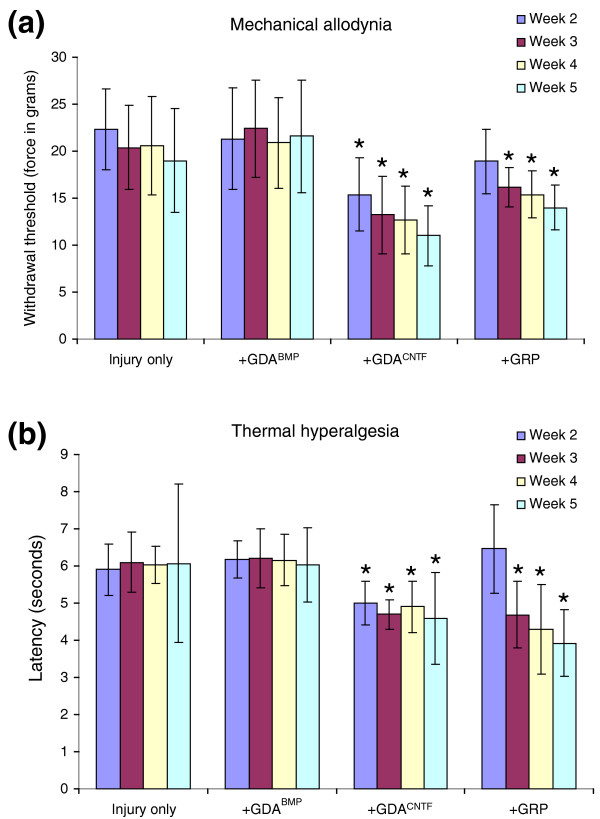
Von Frey filament and hot-plate analysis of mechanical and thermal allodynia. **(a) **Withdrawal threshold of the right front paw to a mechanical stimulus (force in grams). Measurements were made on GRP- or GDA-transplanted and injured control animals at 2, 3, 4 and 5 weeks after dorsolateral funiculus injury/transplantation. **(b) **Latency (in seconds) to paw withdrawal from a heat source. Note that injury alone and GDA^BMP ^transplantation do not induce statistically significant mechanical or thermal allodynia at any time point. However, the mechanical threshold and latency to withdrawal from a heat source are significantly lower in GDA^CNTF^- and GRP-transplanted rats beginning at 2 and 3 weeks, respectively, post-injury/transplantation. Asterisks denote a statistical difference from time-matched control animals (two-way repeated measures, ANOVA, *p *< 0.05). Error bars represent 1 SD.

To test whether transplantation of GDAs^BMP^, GDAs^CNTF ^and GRPs might promote the induction of mechanical allodynia and thermal hyperalgesia in acute spinal cord injuries, initial experiments were carried out to test for increases in mechanical and thermal sensitivity in control rats receiving injections of medium into transection injuries of the right-side dorsolateral funiculus at 2, 3, 4, and 5 weeks after injury. Importantly, compared with pre-injury scores, injured medium-injected control rats did not show statistically significant increases in gram force withdrawal thresholds for right-side forepaws in response to application of graded Von Frey filaments at all time points after injury and transplantation (Figure 10a). Similarly, analysis of paw-withdrawal response latencies to an experimental heat source pre- and post-injury revealed no statistically significant induction of thermal hyperalgesia in injured controls at all time points post-injury (Figure 10b). These results enable a direct comparison of the effects of intra-injury transplantation of GDAs^BMP^, GDAs^CNTF ^or GRPs on the induction of mechanical allodynia and thermal hyperalgesia in rats with identical dorsolateral funiculus transection injuries.

Unlike animals that received GDAs^CNTF ^or GRPs, GDA^BMP^-transplanted animals did not show a statistically significant increase in sensitivity to mechanical or heat stimuli at any times (2, 3 and 4 weeks) up to 5 weeks post-injury (Figure 10a,b) compared to pre-injury responses (two-way repeated measures ANOVA, *p *> 0.05). GDA^CNTF^-transplanted animals showed a significant increase in sensitivity to both mechanical and heat stimuli by 2 weeks after injury, an effect that persisted to 5 weeks after injury, the last time point tested (Figure 10a,b). Animals that received intra-injury transplants of undifferentiated GRPs also developed increased sensitivity to both mechanical and heat stimuli, although with a delayed time course. GRP-transplanted animals began to show mechanical allodynia and thermal hyperalgesia at 3 weeks post injury and transplantation, effects that persisted to 5 weeks post-injury (Figure 10a,b).

### GDAs^CNTF ^and GRPs promote sprouting of CGRP c-fibers after spinal cord injury

**Figure 11:**
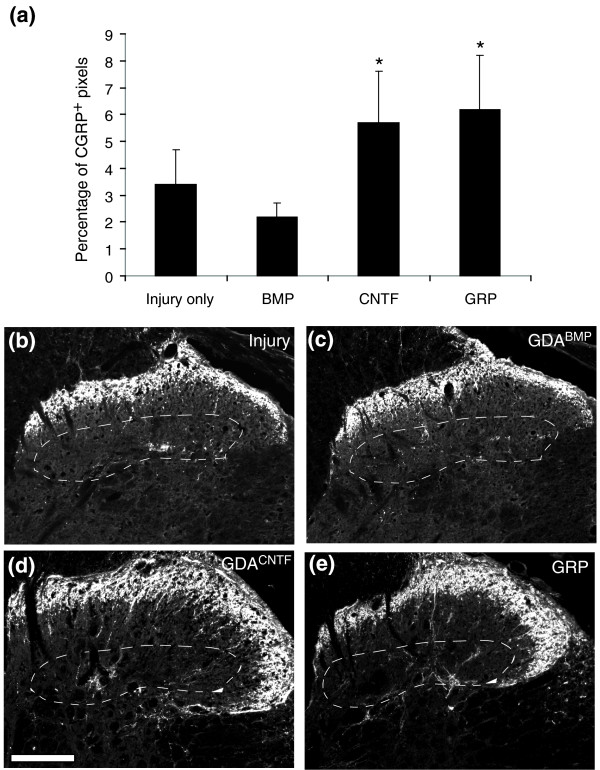
Aberrant CGRP^+ ^c-fiber sprouting into lamina III of GDA^CNTF^- or GRP-transplanted spinal cords that have received dorsolateral funiculus transection injuries. The density of pixels within images of lamina III of the right-side dorsal horn caudal to the injury and transplantation site in GDA- or GRP-transplanted, or injury-only control animals is presented as the average percentage of CGRP^+ ^pixels per total pixels (area) of lamina III. **(a) **Averages of 5.7% and 6.2% of the total pixels in lamina III were CGRP^+ ^in GDA^CNTF^- and GRP-transplanted spinal cords, respectively. In contrast, only 2.2% and 3.4% of lamina III pixels were CGRP^+ ^in GDA^BMP^-transplanted and injury-only spinal cords. The asterisk indicates significant difference from both control injury-only and GDA^BMP^-transplanted groups (two-way repeated measures ANOVA, *p *< 0.05). Error bars represent 1 SD. **(b-e) **Sample images of sections labeled with anti-CGRP antibodies from rats transplanted at the spinal C6 level: (b) control, (c) GDA^BMP^, (d) GDA^CNTF ^and (e) GRP. Area enclosed with a dashed line in (b-e) indicates lamina III. Note the increased density of CGRP^+^ immunoreactivity within lamina III of the dorsal horn of (d) GDA^CNTF^- and (e) GRP-treated spinal cords compared to (b) control injured and (e) GDA^BMP^-treated spinal cords. Scale bar 200 μm.

Previous studies have shown a correlation between sprouting of calcitonin-gene-related peptide (CGRP) immunoreactive nociceptive c-fibers within lamina III of the dorsal horn and the development of neuropathic pain after spinal cord injury [30]. To assay for this, we carried out a comparative quantitative analysis at 5 weeks post-injury of the density of CGRP immunoreactivity in lamina III of the dorsal horn at spinal level C6 ipsilateral to injury sites in media-injected injured controls, and GDA^BMP^-, GDA^CNTF^- or GRP-transplanted animals. Notably, the GDA^BMP^-transplanted animals showed no statistically significant change in the density of CGRP-positive c-fibers compared with the control injured animals (Figure 11). This result correlated with the absence of statistically significant mechanical allodynia and thermal hyperalgesia in GDA^BMP^-treated animals compared with uninjured controls.

In contrast, significant increases in the density of CGRP immunoreactivity were found in animals that received intra-injury transplants of GDAs^CNTF^ or GRPs. Comparison of the density of CGRP immunoreactivity in GDA^CNTF^-, GRP- and GDA^BMP^-transplanted cords revealed 2.6- and 2.9-fold increases for GDA^CNTF^- and GRP-treated cords, respectively, above levels in GDA^BMP^-transplanted cords at 5 weeks post-injury (Figure 11).

These results demonstrate that transplantation of GDAs^CNTF ^or GRPs, but not of GDAs^BMP^, into spinal cord injuries induces both mechanical allodynia and thermal hyper-algesia, effects that correlated with the relative densities of CGRP immunoreactive c-fibers in dorsal horn lamina III. Collectively, these results show that pain syndromes are not a necessary consequence of astrocyte transplantation or of the generation of astrocytes from transplanted precursor cells at sites of spinal cord injury, but instead indicate that the generation of specific subtypes of astrocyte, such as GDAs^CNTF^, is responsible for this adverse effect.

## Discussion

This study provides several new discoveries related to the treatment of traumatic spinal cord injury by cell transplantation. We show for the first time that different types of astrocytes derived from the same population of embryonic glial precursor cells have markedly different effects on repair and functional recovery when transplanted into the injured adult spinal cord. Transplantation of GDAs^BMP ^promoted axon regeneration, neuroprotection and robust recovery of function. In sharp contrast, transplantation of GDAs^CNTF ^or of undifferentiated GRPs did not provide any of these beneficial effects (see Table 1). Moreover, transplantation of GDAs^CNTF ^or undifferentiated GRPs resulted in both mechanical allodynia and thermal hyperalgesia, problems that were not caused by transplantation of GDAs^BMP^. Our study provides further evidence that astrocytes derived from BMP-treated GRPs are a particularly promising population of cells for CNS repair and provide the first identification of a specific glial cell type – GDAs^CNTF ^– that can induce pain-related syndromes following transplantation into the injured spinal cord.

**Table 1:** Summary of GDAs^BMP^, GDAs^CNTF ^and GRPs effects on spinal cord injury repair and allodynia

Effect	GDAs^BMP^	GDAs^CNTF^	GRPs
Promote axon growth across spinal cord injury	+++	-	-
Promote locomotor recovery	+++	-	-
Suppress atrophy of injured red nucleus neurons	+++	-	-
Induce mechanical and thermal allodynia	-	+++	+++
Promote sprouting of CGRP^+ ^axons in lamina III	-	+++	+++
Align host astrocytes at injury site	+++	-	-
Transiently suppress host CSPG expression	+++	+++	+++
Express inhibitory CSPGs within injury site	-	+++	+++
Express CSPGs *in vitro*	+	+++	+++

### Controlled differentiation of glial precursors and spinal cord repair

The remarkably consistent and robust support of endogenous axon regeneration, neuroprotection and functional recovery provided by transplantation of GDAs^BMP ^in our previous [14] and present studies and the equally consistent failure of GRP transplantation to provide these benefits clearly show that controlled differentiation of glial precursors prior to transplantation to acute spinal cord injuries can result in significantly better outcomes. This hypothesis is consistent with previous studies showing failures of transplanted GRPs to promote axon growth [31] or functional recovery after spinal cord injury unless combined with additional treatments. Genetic manipulation of GRP cells to express the multifunctional-neurotrophin D15A before transplantation [32], or their transplantation in combination with neuron-restricted precursors (NRPs) [33], resulted in some locomotor recovery, with both studies showing improvements of approximately 2.5 points on the Basso, Beattie, and Bresnahan (BBB) open-field locomotor test. However, even the NRP/GRP-treated rats that had shown improved BBB scores failed to show a statistically significant improvement after grid-walk analysis [33]. As the NRP/GRP transplants were carried out in animals with contusion spinal cord injuries, the outcomes cannot be directly compared with our current studies, but do indicate the importance of conducting future experiments to compare the effects of NRP/GRP versus GDA^BMP ^transplantation in promoting recovery from both transection and contusion spinal cord injuries.

A striking and somewhat unexpected result of our study is that the two populations of astrocytes derived by pre-differentiation of embryonic spinal cord GRPs by two classical astrogenesis signaling pathways had completely opposite effects on axon regeneration, neuroprotection, functional recovery and neuropathic pain after transplantation, clearly demonstrating that not all astrocytes that can be derived from glial precursors are beneficial for CNS repair. Previous studies have shown functional differences in astrocytes from different regions of the CNS in respect of promotion of neurogenesis, neurite outgrowth, or promotion of axonal versus dendritic specialization [34-37]. Our current study, however, is the first to demonstrate that astrocytes generated by exposing the same precursor cell to different signaling agents have markedly different effects when transplanted into acute spinal cord injuries.

### Factors regulating GRP differentiation into beneficial astrocytes

In the light of our new findings, the question arises as to whether any astrocyte generated by exposure of precursor cells to BMP would be suitable for use in repair of spinal cord injuries. This may not be the case, however, as exposure of O-2A progenitors cells (a type of glial progenitor that arises later in development than GRP cells) to BMP generate astrocytes with a phenotype that appears to be like that of GDAs^CNTF ^[38], and BMP treatment of acute spinal cord injuries can promote scar formation [39]. These findings suggest that glial precursors isolated from later stages of neural development may not be able to generate beneficial GDA^BMP^-like astrocytes in response to BMP.

### Endogenous GDA^CNTF^-like astrocytes in the injured CNS

It will be of great interest to determine whether the Olig2^+^/GFAP^+^ cells generated in spinal cord injuries (Figure 3c), cerebral cortex stab injuries [21] and in rodent models of experimental autoimmune encephalomyelitis [20] are astrocytes generated from endogenous O-2A progenitor cells, and thus represent the long-sought *in vivo *counterpart of the type-2 astrocytes generated from these progenitor cells *in vitro *[40]. Our findings that GDAs^CNTF ^can become Olig2-negative after transplantation shows that the phenotype of such cells can be labile *in vivo*, as has also been seen in studies of Olig2^+ ^astrocytes during CNS development [41]. This raises the possibility that the search for endogenous GDA^CNTF^-like cells may have to be conducted with a variety of markers, at multiple time points after injury. Nonetheless, the fact that astrocytes within adult CNS scar tissue share many characteristics with GDAs^CNTF^, such as poor support of axon growth and expression of Olig2 and inhibitory CSPGs, supports the hypothesis that they are functionally similar.

### GDAs and suppression of axon-growth-inhibitory scar formation

Our study sheds new light on the role of GDA-mediated suppression of glial scar formation in supporting axon regeneration across acute spinal cord injuries. Logic dictates that improved alignment of host tissue can increase the efficiency of axon growth into and out of a site of injury by creating a shorter, less tortuous path for axons to follow. Neurocan and NG2 are axon-growth-inhibitory CSPGs [42,43] that are upregulated at sites of spinal cord injury [18,44,45] and whose suppression has been shown to correlate with the ability of adult sensory axons to cross acute spinal cord injuries [46]. In light of our previous finding that transplantation of GDAs^BMP^ to acute dorsal column transection injuries resulted in a remarkable alignment of host astrocytes within injury margins and a transient suppression of neurocan and NG2 [14], we proposed that these effects played significant roles in promoting axon growth across GDA^BMP^-bridged spinal cord injuries.

Our current study shows that transplanted GDAs^CNTF ^and GRP cells promote the suppression of neurocan and NG2 in host tissues to an extent comparable to that previously observed for GDAs^BMP ^at 4 days post-transplantation [14]; nevertheless, they completely fail to align host astrocytes or support the regeneration of ascending dorsal column axons across sites of injury. A suppression of CSPG expression combined with a failure of axon regeneration has also previously been shown after GRP transplantation into acute spinal cord injuries [31]. Although these results do not rule out the possibility that suppression of CSPGs in host tissues may play an important role in the ability of GDAs^BMP ^to promote axon growth across sites of injury, they do show that such suppression is not by itself sufficient to promote axon growth. It may be that despite being able to suppress expression of axon-growth-inhibitory CSPGs, GDAs^CNTF ^and GRPs transplanted in acute spinal cord injuries fail to actively support axon growth and/or express molecules themselves that actively inhibit it. These concepts are supported by the expression of neurocan and NG2 by transplanted GDAs^CNTF ^and GRPs, a result that was not observed for transplanted GDAs^BMP ^[14]. The low-level expression of axon-growth-inhibitory CSPGs by GDAs^BMP ^compared with GDAs^CNTF^, both *in vitro *and within spinal cord injuries, is one potential mechanism that might account for the clear difference in the ability of these astrocytes to support axon growth.

### Precursor-derived astrocytes, spinal cord injury and neuropathic pain

Some of the most important results of our experiments concern the ability of both GDAs^CNTF ^and GRP transplants to cause mechanical allodynia and thermal hyperalgesia. Two studies of NSC transplantation to acute traumatic spinal cord injury sites in adult rats showed similar degrees of both mechanical and thermal forelimb allodynia [15,16]. That suppressing the differentiation of the transplanted NSCs to astrocytes prevented the onset of allodynia [15] could be interpreted to mean that all astrocytes generated from precursor cells have the capacity to promote allodynia. It was therefore crucial to determine whether the onset of allodynia after spinal cord injury is a problem that applies generally to the transplantation of astrocytes and astrocyte precursors. Our finding that mechanical allodynia and thermal hyperalgesia are caused by transplantation of GRPs and GDAs^CNTF ^– but not of GDAs^BMP ^– shows that only specific types of astrocytes or glial precursors cause these adverse outcomes.

Aberrant sprouting of CGRP-positive c-fibers has been shown to correlate with the onset of neuropathic pain after spinal cord injury [30]. We found a doubling of the density of CGRP-positive c-fibers within lamina III of the dorsal horns of injured spinal cords receiving GDA^CNTF ^or GRP transplants, an effect that was also correlated with neuropathic pain after transplantation of neural precursor cells into acute spinal cord injuries [15,16]. Interestingly, a reduction in allodynia and sprouting of CGRP^+^ c-fibers has been observed after transplantation of mixed NRP/GRP populations to spinal cord injuries [33]; however, the effects of GRP transplantation alone on allodynia were not tested in that study. Whether this benefit of combined NRP/GRP transplantation reflects suppression of the generation of GDA^CNTF^-like astrocytes at the site of injury is an interesting question for the future.

It has long been a concern that therapies designed to promote axon growth after spinal cord injury would result in sprouting of CGRP^+ ^c-fibers and the induction of neuropathic pain. Our results show, however, that GDAs^BMP ^have the remarkable ability to promote functional recovery without inducing pain, and promote axon regeneration without promoting aberrant sprouting of CGRP^+ ^c-fibers. These results also show for the first time that two different types of precursor-derived astrocytes can have markedly different effects on the growth of different types of sensory axons.

### Glial activation and neuropathic pain after spinal cord injury

Glial activation within the injured adult spinal cord is thought to have an important role in the development and maintenance of neuropathic pain [47,48]. A current model of glial cell function in neuropathic pain hypothesizes that injury-activated microglia are critical for initiation of enhanced pain perception via activation of astrocytes, and that activated astrocytes and microglia are also involved in the maintenance of neuropathic pain after traumatic spinal cord injury [49]. It has been shown that increases in spinal cord astrocytic GFAP expression following peripheral nerve injury correlates with the development of neuropathic pain [50] and that specific activation of microglia and astrocytes in the adult rat spinal cord is sufficient to promote neuropathic pain [51]. Whether transplanted GDAs^BMP ^or GDAs^CNTF ^can alter the activation state of microglia, whether these cells respond differently to the presence of activated microglia in an acute spinal cord injury, or whether transplantation of GDAs^CNTF ^bypasses any requirement for activated microglia to initiate neuropathic pain, are all presently unknown and will be the subject of future investigations.

### Neuropathic pain, glial scar formation and gp130 receptor activation

The results reported here may also prove relevant to a better understanding of the role of gp130 agonists in promoting glial scar formation and neuropathic pain syndromes. In addition to its interactions with CNTF, the gp130 protein is a shared receptor for several related cytokines, including leukemia inhibitory factor (LIF) and interleukin-6 (IL-6, which has tertiary structure homology with CNTF) [52]. In some contexts, these agents may have beneficial effects on the injured nervous system, such as promoting oligodendrocyte generation and survival as well as neuronal protection [53-59]. Our results show, however, that exposure of glial precursors to the gp130 agonist CNTF results in the generation of astrocytes that are poorly supportive of axon growth and promote pain when transplanted into spinal cord injuries. CNTF, LIF and IL-6 are known to be upregulated at sites of spinal cord injury [60-65] and it is possible that some or all of these factors may also drive the differentiation of local endogenous glial precursors to a GDA^CNTF^-like phenotype (Olig2^+^/GFAP^+^) that contributes to the formation of axon-growth-inhibitory scar tissue. Our finding of endogenous Olig2^+^/GFAP^+ ^astrocytes within the margins of control untreated spinal cord injuries lends at least preliminary support to this hypothesis. Recent experiments showing that blocking of the gp130 receptor suppressed scar formation and improved functional recovery after spinal cord injury [66], and that specific inhibition of CNTF induction of astrocyte differentiation within transplanted fetal tissue improved axon growth across acute spinal cord injuries [67], also support this hypothesis. Future experiments will determine whether blocking CNTF or gp130 receptor activity in spinal injuries or after transplantation of undifferentiated glial precursors will increase axon growth across the injury and suppress the onset of neuropathic pain.

## Conclusion

The results reported here lend significant new support to our hypothesis that pre-differentiation of glial precursor cells into a specific population of astrocytes such as GDAs^BMP ^before transplantation into spinal cord injuries results in significantly better outcomes, and they also provide further evidence that GDAs^BMP ^are a particularly promising cell type for promoting CNS repair. We have also provided the first identification of a specific glial cell type – GDAs^CNTF ^– that is capable of inducing pain-related syndromes following its transplantation into the injured spinal cord. This clearly demonstrates that not all astrocytes that can be derived from embryonic glial precursors have beneficial effects in spinal injuries. As gp130 agonists are of broad interest as inducers of astrocyte reactivity after injury to the CNS, our present findings are of particular relevance to the future study of gp130 agonists and glial precursors in CNS scar formation and onset of allodynia. The generation of a pain syndrome is one of the most adverse outcomes that could result from cell transplantation therapy for spinal cord injury [68-71], rivaled only by loss of remaining function or increased mortality. Our findings demonstrate that a better understanding of the origins and functional properties of different subpopulations of astrocytes is required if we are to safely utilize CNS stem or progenitor cell transplantation for treating the injured or diseased adult CNS.

## Materials and methods

### Isolation of GRPs and generation of GDAs

A2B5^+ ^GRPs were isolated by fluorescence activated cell sorting (FACS) of dissociated cell suspensions from spinal cords of embryonic day (E)13.5 transgenic Fischer 344 rat embryos expressing the gene for human placental alkaline phosphatase (hPAP) under the control of the ROSA26 promoter (TgN(R26ALPP)14EPS) [72]. GRPs were maintained on a fibronectin/laminin substrate at 4 × 10^3 ^to 2 × 10^4 ^cells/cm^2 ^in Dulbecco's modified eagle medium (DMEM)/F12 Sato-medium supplemented with 10 ng/ml basic fibroblast growth factor (bFGF). Passage number, days *in vitro*, cell density and media conditions were tightly controlled for experimental replicates. To differentiate GRPs before transplantation, 10 ng/ml of human recombinant BMP-4 (R&D Systems) or 10 ng/ml human recombinant CNTF (Peprotech) were added to the culture media for 7 days to differentiate them into astrocytes – GDAs^BMP ^(A2B5^-^/GFAP^+^) and GDAs^CNTF^ (A2B5^+^/GFAP^+^), respectively. For *in vitro *induction experiments, GRPs were seeded at 5,000 cells/cm^2 ^on a fibronectin/laminin substrate in DMEM/F12 Sato-medium with 10 ng/ml bFGF. After 18 h, cell culture conditions were switched as indicated and cells were allowed to differentiate into astrocytes for up to 7 days. Medium was changed every 2 days. Parallel cultures were used for Western blot and immunofluorescent analysis.

### *In vitro *immunofluorescence

Cells grown on fibronectin/laminin-coated glass coverslips were fixed for 5 minutes in 2% formaldehyde, rinsed and blocked using 5% normal goat serum in Hanks balanced salt solution (HBSS) with Hepes pH 6.8. For Olig2 and GFAP labeling, cells were permeabilized using 0.1% Triton-X100 in phosphate buffered saline (PBS) for 15 minutes. Anti-Olig2 (1:4000, Chemicon) and anti-GFAP (1:400, Cell Signaling) were incubated at 4°C for 18 h. Anti-NG2 (Chemicon, 1:2000) staining was performed on live cells in growth medium for 30 minutes prior to fixation with formaldehyde. Fluorescently labeled, secondary anti-Ig antibodies (Alexa 488 and 568 conjugates, Invitrogen) were used at a 1:2000 dilution for 1 h at room temperature. Coverslips were mounted on glass slides with ProLong Gold and viewed using a Nikon 80i microscope equipped with a Spot RT camera. Monochrome images of parallel samples were captured using identical exposure times and gain settings, and merged as pseudo-colored images. Both BMP- and CNTF-induced GDAs were uniformly immunoreactive for human alkaline phosphatase *in vitro*.

### Western blot analysis

After treatment of cultures for 5 days with conditions as indicated, PBS-washed cells were harvested in XDP buffer (1% Triton X100, 0.5% sodium deoxycholate in PBS pH 7.2) supplemented with Complete Mini Protease Inhibitor Cocktail (Roche). The protein concentration of cleared lysates was determined using the Biorad D_C _protein assay. Samples (25 μg of protein per sample) were fractionated using NuPage 4–12% gradient gels (Invitrogen) and then transferred to polyvinylidene difluoride (PVDF) membranes (Perkin Elmer). Membranes were blocked in 5% non-fat dry milk in Tris-buffered saline containing 0.1% Tween-20 (Sigma) and then incubated with primary antibodies at 4°C for 18 h. Antibodies and dilutions used: NG2 (Chemicon, 1:1000), anti-phosphacan (Developmental Studies Hybridoma Bank, 1:1000), β-tubulin (Santa Cruz Biotechnology, 1:1000). Horseradish-peroxidase-conjugated anti-mouse (PerkinElmer) or anti-rabbit (Invitrogen) antibodies were applied to washed blots and visualized using Luminol reagent (Santa Cruz Biotechnology) and Kodak X-OMAT LS X-ray film. Film was developed using a Kodak X-OMAT 3000RA processor. Densitometric analysis of scanned film images was performed using NIH Image-J software. Expression levels of phosphacan (320–340 kDa band) and NG2 (270–300 kDa band), respectively, were normalized for each sample to β-tubulin (52 kDa) expression. All Western blot experiments were conducted in triplicate and results were compared using the Student's *t*-test, *p *< 0.05.

### Homogeneity of cell populations for transplantation

To confirm cell phenotype and homogeneity before transplantation, small volumes of cell suspensions were plated onto glass coverslips and labeled with A2B5 and anti-GFAP antibodies. GRP cell suspensions occasionally contained a small number (average of 2.1%) of A2B5^+^/GFAP^+ ^cells, and GDA^CNTF ^cell suspensions included a small number (average 1.3%) of A2B5^+^/GFAP^- ^cells. To ensure that GDA^BMP ^suspensions for transplantation did not contain undifferentiated GRPs or cells with the phenotype of CNTF-induced astrocytes (A2B5^+^/GFAP^+^), potential contaminating cell types were removed from the suspension by immunopanning with the A2B5 antibody. For transplantation, GRPs or GDAs were suspended in HBSS at a density of 30,000 cells/μl.

### Spinal cord injury models and cell transplantation

Adult female Sprague Dawley rats (3 months old, Harlan) were used in all *in vivo *spinal cord injury experiments (see Table 2 for numbers of rats used per experiment) and were anesthetized by injection of a cocktail containing ketamine (42.8 mg/ml), xylazine (8.2 mg/ml), and acepromazine (0.7 mg/ml). For dorsal column injuries (Figure 1a–c), the right-side dorsal column was unilaterally transected between cervical vertebrae 1 and 2 using a 30-gauge needle as a blade (see also [18,25,46]). Injuries extended to a depth of 1 mm and extended laterally 1 mm from the midline. For rubrospinal tract injuries, unilateral transections of the right-side dorsolateral funiculus including the rubrospinal pathway were conducted at the C3/C4 spinal cord level with Fine Science Tools micro-scissors. Injuries extended to a depth of 1 mm and extended medially 1 mm from the lateral pial surface of the spinal cord (Figure 1d). Transection spinal cord injuries were used instead of contusion injuries in order to minimize axon sparing and permit more accurate quantification of axon growth across injury sites bridged with GDA^CNTF^, GDA^BMP ^or GRPs. The use of an intervertebral surgery approach in combination with discrete transection injuries of the dorsolateral funiculus also results in highly consistent deficits in grid-walk locomotor performance and atrophy of red nucleus neurons [14].

**Table 2:** Numbers of animals per experimental group *in vivo*

Spinal cord injury model	Details	Time points	Control injury	+GDA^BMP^	+GDA^CNTF^	+GRP
Dorsal column injury	Analysis of scar formation and transplanted cell phenotype	4 days	4	4	4	4
		8 days	4	4	4	4
Dorsal column injury	Analysis of endogenous axon growth	8 days	-	5	5	5
Dorsal column injury	Analysis of axon growth from transplanted GFP^+ ^sensory neurons	8 days	4	-	4	-
Dorsolateral funiculus injury	Analysis of locomotor recovery, allodynia and neuroprotection	5 weeks	9	9	9	9

A total of 6 μl of GDA^CNTF^, GDA^BMP ^or GRP suspensions (30,000 cells/μl; 180,000 cells total) per animal were acutely transplanted into six different sites in dorsal column injuries; that is, two injections each into medial and lateral regions of the rostral and caudal injury margins, and two injections into medial and lateral regions of the injury center (Figure 1b). All dorsal column injury experiments were conducted in the absence of immunosuppressants. Transplants of either GDAs^BMP^, GDAs^CNTF ^or undifferentiated GRPs were injected in an identical pattern into injuries of the dorsolateral funiculus and a total of 6 μl of GDA or GRP cell suspension (30,000 cells/μl; 180,000 cells) injected per injury site. Control injured rats were injected with 6 μl HBSS. All control or cell transplanted rats in the dorsolateral funiculus injury groups were given daily injections of cyclosporine (1 mg/100 g body weight) beginning the day before injury/transplantation through to experimental endpoints.

### Adult DRG neuron transplantation

Single-cell suspensions of adult mouse sensory neurons were prepared from 10–12-week-old transgenic mice expressing the gene for EGFP [73] as previously described [25,46,74]. No growth factors were added to the neuron suspension. Five hundred nanoliters of the neuron suspension (approximately 1,500 neurons/μl) were acutely microtransplanted into dorsal column white matter approximately 500 μm caudal to the injury site (Figure 1c).

### Histology

At 4 days, 8 days and 5 weeks post-surgery animals were deeply anesthetized and transcardially perfused with 0.1 M PBS followed by 4% paraformaldehyde in 0.1 M PBS. Dissected spinal cords were cryoprotected in a 30% sucrose/PBS solution at 4°C overnight. Tissue was embedded in optimal cutting temperature (OCT) medium (Sakura Finetek) and quickly frozen. Serial 25-μm-thick frozen sections were cut in the sagittal plane and air dried onto gelatin-coated glass slides. All tissue sections were washed in PBS, blocked with 4% normal goat serum in solution with 0.1% Triton/PBS for 30 minutes, then incubated with appropriate primary antibodies in the blocking solution overnight at 4°C. Secondary antibody incubations were for 45 minutes at room temperature.

The following primary antibodies were used: monoclonal anti-GFAP (Sigma) and polyclonal anti-GFAP (Sigma); polyclonal anti-NG2 (Chemicon); monoclonal anti-neurocan (clone 1F6, Developmental Studies Hybridoma Bank); polyclonal anti-GFP (Molecular Probes); monoclonal anti-hPAP (Sigma); polyclonal anti-hPAP (Fitzgerald); polyclonal anti-Olig2 (Chemicon); polyclonal anti-CGRP (Chemicon). Cy5, Cy2 (Jackson), Alexa-488 and Alexa-594 (Molecular Probes) conjugated secondary antibodies were used to visualize primary antibody binding. All secondary antibodies were pre-absorbed against rat serum. To control for nonspecific secondary antibody binding, adjacent sections were also processed as described above without primary antibodies. Some sections were counterstained with DAPI to show nuclei. Labeled sections were examined and imaged using a Zeiss Observer Z1 fluorescence light microscope or a Zeiss 510 Meta confocal microscope. Antigen co-localization and cellular associations were determined with Zeiss Confocal image analysis software. Spinal cord white matter rostral to the injury site is shown to the left in all figures with images of sections cut in the sagittal plane.

### Tracing and quantification of endogenous ascending dorsal column axons

In the dorsal column injury model, ascending endogenous axons were traced by injection of 10% biotinylated dextran amine in sterile PBS (BDA, Molecular Probes) at 8 days prior to an experimental endpoint. BDA tracer was injected to a depth of 0.5 mm into the right-side, cuneate and gracile white matter at the C4/C5 spinal level (Figure 1c). For histological analysis of BDA-labeled axons, 25-μm serial sagittal sections were collected and processed for immunohistochemistry as described above. BDA was visualized by incubating tissue sections with the Vectastain ABC solution (Vector Labs), and further intensified with the Tyramide-Alexa 488 reagent (Molecular Probes).

For quantification of axon regeneration, the number of BDA-labeled axons was counted in every third tissue section spanning the medial-lateral extent of dorsal column injury sites at the following locations: 0.5 mm caudal to the injury; directly at the injury center; 0.5 mm, 1.5 mm and 5 mm rostral to the injury site; and within the dorsal column nuclei. To control for differences in axon tracing/labeling efficiency between animals, the numbers of BDA-labeled axons counted within the injury center and at all rostral sites were normalized to the number of BDA-labeled axons detected 0.5 mm caudal to the injury site for each tissue section examined. The normalized values from each tissue section for each separate animal (control, GRP-, GDA^BMP^- and GDA^CNTF^-transplanted rats) were averaged to generate values for each animal. The values for each animal (*n* = 5 per group) were then averaged and displayed graphically. ANOVA or *t*-tests were performed as appropriate, *p *< 0.01.

### Quantification of CGRP c-fiber sprouting

For quantifying changes in the density of CGRP immunoreactivity in rats that had received right-side dorso-lateral funiculus transection injuries, 20-μm-thick serial cross-sections were labeled with anti-CGRP antibody. Images were captured of the right-side dorsal horn (ipsilateral to the injury/transplantation site) from five randomly chosen sections at the C6 spinal level from five animals in each experimental group. Analysis was conducted at the C6 spinal level because this is the level that maps to the dermatome as tested for forepaw mechanical sensitivity in rats [75]. All images were captured at the same magnification, resolution and exposure time. Using ImagePro image analysis software, lamina III of the dorsal horn was selected as the region of interest and the number of pixels within lamina III that were CGRP-positive was recorded. The total number of pixels within each region of interest was also recorded and used to normalize CGRP pixel counts between sections and thus permit comparison of the density of CGRP immunoreactivity between experimental groups. Data are presented as the average percentage of CGRP-positive pixels per area sampled (number of CGRP-positive pixels divided by the total number of pixels per region of interest) and analyzed by ANOVA followed by Tukey's post test. An ANOVA analysis was also carried out to ensure that the total area sampled between groups was not significantly different (*p *> 0.05).

### Grid-walk behavioral analysis

Two weeks before surgery, rats were trained to walk across a horizontal ladder (Foot Misplacement Apparatus, Columbus Instruments) and only rats that consistently crossed without stopping were selected for experiments. The grid-walk test is a sensitive measure of the ability of rats to step rhythmically and coordinate accurate placement of both fore and hind limbs [27]. For analysis of recovery of locomotor function in GDA^BMP^- and GDA^CNTF^-transplanted versus untreated injured controls, trained rats were randomly assigned to one of three groups: RST injury + GDA^BMP ^+ cyclosporine (*n* = 9); RST injury + GDA^CNTF ^+ cyclosporine (*n* = 9); RST injury + suspension media + cyclosporine (*n* = 9). One day before surgery (baseline) and at 3, 7, 10, 14, 17, 21, 24 and 28 days post-surgery, each rat was tested three times and the number of mis-steps from each trial was averaged to generate a daily score for each animal. Two-way repeated measures ANOVA and Tukey post test (*p *< 0.05) were applied to analyze the data.

### Sensory testing

Mechanical and thermal sensitivity were measured the day before injury/transplantation (baseline) and then at 2, 3, 4 and 5 weeks post-injury. To test for changes in mechanical sensitivity, graded Von Frey filaments (Stoelting) were applied in ascending order to the plantar surface of the right forepaw. The lowest force that caused paw withdrawal accompanied by licking, paw-guarding behavior or vocalization at least three times per five trials was determined to be the mechanical threshold. Thermal sensitivity was tested with the hot-plate analgesia instrument (Stoelting). The temperature of the plate was held constant at 55°C, rats were placed on the plate, and the latency (in seconds) to licking of paws or vocalization was recorded. ANOVA followed by Tukey's post test analysis was applied to determine statistical significance of any change from baseline behavior (*p *< 0.05). Analysis was conducted on the same GDA^BMP^-treated, GDA^CNTF^-treated, and medium-injected control rats with spinal cord injury that were used for grid-walk analysis. An additional group of GRP-transplanted rats with dorsolateral funiculus injuries (*n* = 9) was also similarly tested for mechanical allodynia and thermal hyperalgesia at times ranging from 2 to 5 weeks after injury/transplantation.

### Quantification of red nucleus neurons

At 5 weeks after injury/transplantation, animals were euthanized and 25-μm serial frozen sections were cut in the coronal plane from the brains of rats that had undergone behavioral analysis. Every third section through the rostro-caudal extent of the red nucleus was stained with 0.2% cresyl violet. Standard, design-based stereology methods (CAST software, Olympus) were used to quantify numbers of neurons in both red nuclei in six out of nine RST-injured rats per group that had received GDA^CNTF^, GDA^BMP^, or GRP transplants or control injections of culture medium. An optical fractionator was applied to left and right side red nuclei from every sixth section. Cell bodies greater than 20 μm in diameter and with characteristic neuronal morphology were counted. The numbers of neurons counted in the left-side (injured) red nucleus were normalized to counts obtained for the uninjured right-side nucleus for each animal. The values for each animal within a group were averaged and displayed graphically. A *t*-test was performed to determine the statistical significance of the difference between the groups (*p *< 0.01).

All procedures were performed under guidelines of the National Institutes of Health and approved by the Institutional Animal Care and Utilization Committee (IACUC) of Baylor College of Medicine, Houston, TX or the IACUC of University of Colorado Health Sciences Center, Denver, CO, or the IACUC of University of Rochester Medical Center, Rochester, NY.

## Additional data files

Additional data file 1 is a figure showing that transplanted GRP cells express neurocan and NG2, but suppress host expression of these molecules at 4 days post transplantation to dorsal column injuries. Additional data file 2 is a figure showing failure of axons to regenerate across GDACNTF-transplanted injuries. Additional data file 3 is a figure showing neuroprotection of injured red nucleus neurons.

## Supplementary Material

Additional data file 1 A figure showing that transplanted GRP cells express neurocan and NG2, but suppress host expression of these molecules at 4 days post transplantation to dorsal column injuries. Click here for file

Additional data file 2 A figure showing failure of axons to regenerate across GDACNTF-transplanted injuries. Click here for file

Additional data file 3 A figure showing neuroprotection of injured red nucleus neurons. Click here for file
